# 接枝多胺聚合物制备大孔阴离子交换色谱介质及其蛋白吸附行为评价

**DOI:** 10.3724/SP.J.1123.2023.11003

**Published:** 2024-04-08

**Authors:** Zeping JIANG, Wang GUO, Ziyang LI, Hengyang HOU, Wendi HUO, Jiayi WANG, Lei MA, Haibo JIN, Yongdong HUANG, Rongyue ZHANG

**Affiliations:** 1.北京石油化工学院新材料与化工学院, 燃料清洁化及高效催化减排技术北京市重点实验室, 北京 102617; 1. College of New Materials and Chemical Engineering, Beijing Institute of Petrochemical Technology, Beijing Key Laboratory of Fuel Cleanliness and Efficient Catalytic Emission Reduction Technology Beijing 102617, China; 2.中国科学院过程工程研究所生化工程国家重点实验室, 北京 100190; 2. State Key Laboratory of Biochemical Engineering, Institute of Process Engineering, Chinese Academy of Sciences, Beijing 100190, China

**Keywords:** 大孔聚合物微球, 阴离子交换色谱介质, 接枝法, 蛋白结合容量, macroporous polymer microspheres, anion exchange chromatographic media, grafting, binding capacity of proteins

## Abstract

以环氧活化聚甲基丙烯酸酯大孔微球(FastSep-epoxy)为基质,通过接枝聚(烯丙基胺)(PAA)制备了大孔阴离子交换色谱介质(FastSep-PAA)。考察了介质的合成条件对离子交换容量(IC)及蛋白结合容量的影响,发现IC随PAA浓度、反应时间和溶液pH的增加均表现为增长趋势;同时结合蛋白吸附容量的变化选择了最优合成条件。通过扫描电子显微镜观察介质的表面形貌,发现其孔隙连通性较好,且接枝前后介质孔道结构无明显变化,PAA配基密度对介质结构无明显影响。此外,通过压汞法和氮气吸附法测定接枝前后不同介质的孔径尺寸和孔径分布情况,并考察该类介质的孔径与蛋白吸附行为的关系,发现其蛋白结合容量未出现随介质孔径尺寸增加而显著下降的现象,且孔径尺寸增加更有利于蛋白在介质内部传质。在126 cm/h的流速下FastSep-PAA介质的原始孔径(即FastSep-epoxy的孔径)为400 nm时的蛋白动态结合容量(DBC)最高(70.3 g/L),该孔径下介质比表面积大,蛋白可吸附位点较多;原始孔径为700 nm及以下的介质蛋白DBC均随流速增加而均有一定下降;原始孔径为1000 nm的介质蛋白DBC几乎不受流速影响,流速由126 cm/h增加至628 cm/h时蛋白DBC仅下降3.5%,且在628 cm/h的流速下能保持57.7 g/L,蛋白分子在该孔径下的扩散速率受限较小,传质性能优异,这一特性表明该类大孔聚合物阴离子交换色谱介质在蛋白高通量分离中具有较大的优势。

## Abstract (en)

The macroporous anion exchange chromatographic medium (FastSep-PAA) was prepared through grafting polyallylamine (PAA) onto polyacrylate macroporous microspheres (FastSep-epoxy). The effects of the synthesis conditions, including the PAA concentration, reaction time, and reaction solution pH, on the ion exchange (IC) of the medium were investigated in detail. When the PAA concentration, reaction time, and reaction solution pH were increased, the IC of the medium increased, and optimal synthesis conditions were then selected in combination with changes of protein binding capacity. A scanning electron microscope was used to examine the surface morphology of the medium. The medium possessed high pore connectivity. Furthermore, the pore structure of the medium was preserved after the grafting of PAA onto the macroporous microspheres. This finding demonstrates that the density of the PAA ligands does not appear to have any discernible impact on the structure of the medium; that is, no difference in the structure of the medium is observed before and after the grafting of PAA onto the microspheres. The pore size and pore-size distribution of the medium before and after grafting were determined by mercury intrusion porosimetry and the nitrogen adsorption method to investigate the relationship between pore size (measured in the range of 300-1000 nm) and protein adsorption. When the pore size of the medium was increased, its protein binding capacity did not exhibit any substantial decrease. An increase in pore size may hasten the mass transfer of proteins within the medium. Among the media prepared, that with a pore size of 400 nm exhibited the highest dynamic-binding capacity (DBC: 70.3 g/L at 126 cm/h). The large specific surface area of the medium and its increased number of protein adsorption sites appeared to positively influence its DBC. When the flow rate was increased, the protein DBC decreased in media with original pore sizes of less than 700 nm. In the case of the medium with an original pore size of 1000 nm, the protein DBC was independent of the flow rate. The protein DBC decreased by 3.5% when the flow rate was increased from 126 to 628 cm/h. In addition, the protein DBC was maintained at 57.7 g/L even when the flow velocity was 628 cm/h. This finding reveals that the diffusion rate of protein molecules at this pore size is less restricted and that the prepared medium has excellent mass-transfer performance. These results confirm that the macroporous polymer anion exchange chromatographic medium developed in this study has great potential for the high-throughput separation of proteins.

生物技术的快速发展和人们近年来对生物药物需求的增加,促进了生物制药规模的迅速增长。生物制药上游制备规模的增加对下游的分离纯化技术提出了新的挑战,色谱技术作为当前主流的分离纯化技术^[1]^,色谱介质的性能起到决定性作用,其中高通量是色谱介质的重要参数^[2]^。当前常用的色谱介质多以琼脂糖为基质,其本身质地软(耐压< 0.3 MPa)、平均孔径小(30~50 nm)^[3]^,在高通量分离纯化应用中受到一定限制。为弥补琼脂糖基质色谱介质的这一不足,近年来以有机聚合物为基质的色谱介质受到越来越多的关注,如以聚苯乙烯^[4]^和聚丙烯酸酯类^[5]^微球为基质的色谱介质机械强度高,耐压性可高达10 MPa^[6]^,同时其孔径调控范围广(100~1000 nm)^[7]^,能更好地满足高通量分离纯化的需求。

聚合物基质的色谱介质在机械强度和孔径尺寸方面弥补了常规琼脂糖色谱介质的不足,但因为其本身孔径尺寸大、比表面积低等特点,致使其蛋白结合容量低^[8]^,尤其对于大孔聚合物色谱介质来说更是如此。为提高介质吸附容量,在微球表面接枝聚合物配基是常用策略,利用该类分子的功能基团数目多、分子构象多变等特性,可提高蛋白分子的捕获效率。如本研究小组曾以支化的聚乙烯亚胺(PEI)为配基^[9]^制备了阴离子交换色谱介质,PEI分子具有多个氨基基团,增加了大孔聚丙烯酸酯微球表面的氨基数量,同时PEI长链分子通过“链传递”机理^[10]^进一步提高了蛋白大分子在介质内部的传质,从而增加蛋白的结合容量和传质速率。聚(烯丙基胺)(PAA)作为聚合物配基,其具有直链结构,所带氨基均为伯胺基团,这较PEI配基的支链结构更为简单,所带氨基种类也更为单一,因此更适合用于对阴离子交换配基的吸附与色谱行为进行系统研究^[11⇓⇓⇓⇓⇓⇓-18]^。目前该配基在琼脂糖类色谱介质上已有应用,孙彦课题组曾对不同相对分子质量的PAA配基接枝介质的吸附与色谱行为进行系统研究^[19⇓-21]^,其中17.5 kDa的PAA配基获得了更高的吸附容量与传质性能,其蛋白结合容量比接枝PEI的介质提高20%以上,远超其他类型的阴离子交换色谱介质。但以大孔聚合物微球为基质的该类色谱介质的研究目前较少,其制备方法、配基类型、蛋白吸附行为等研究内容有待深入探究。

本工作以大孔聚丙烯酸酯类微球为基质,通过“ grafting to ”方法接枝PAA配基制备了大孔阴离子交换色谱介质(FastSep-PAA),研究了以PAA为配基的阴离子交换色谱介质的制备方法,探讨了配基密度、孔径尺寸与蛋白结合容量的关系,为该类聚合物色谱介质的制备和应用提供一定的理论参考。

## 1 实验部分

### 1.1 仪器与试剂

扫描电子显微镜(SEM),韩国COXEM公司;TG 16台式高速离心机(TMHSC),上海卢湘仪离心机仪器有限公司;全自动比表面及孔径分析仪(BET),金埃谱(南京)科技有限公司;高性能全自动压汞仪(MIP),深圳华普通用科技有限公司;SCG-100型蛋白色谱仪,苏州赛谱仪器有限公司;L5紫外可见分光光度计(UV-Vis),上海仪电分析仪器有限公司;SHA-BA型水浴恒温振荡器(IS),金坛市荣华仪器制造有限公司;E1677垂直混合仪(VM),宁波新芝生物科技股份有限公司。

环氧活化聚甲基丙烯酸酯大孔微球(FastSep-epoxy,其微球表面为环氧基团)定制于北京博尔赛谱生物科技有限公司,牛血清白蛋白(BSA,纯度98%)购于北京百灵威科技有限公司,PAA(分子质量10~20 kDa,纯度98%)购于北京华威锐科化工有限公司,以上试剂使用前均未进一步纯化。

### 1.2 大孔微球表面接枝PAA

大孔阴离子交换色谱介质的制备过程如下:将4.0 g PAA溶解于20 mL去离子水中,用0.5 mol/L NaOH调节pH=12.0,将2.0 g干燥恒重的大孔微球FastSep-epoxy一并加入50 mL离心管中,于垂直混合仪上室温反应12 h,反应完毕后,将制得的微球用去离子水洗涤至中性,于25 ℃真空干燥24 h后室温存放备用,将其命名为FastSep-PAA(依据原料微球的厂家标示孔径,分别命名为FastSep-PAA-300、400、500、700、1000)。

### 1.3 FastSep-PAA容量

#### 1.3.1 离子交换容量(IC)

量取10 mL FastSep-PAA加入色谱柱中,将50 mL 1 mol/L NaOH加入色谱柱内,自然流尽后用去离子水洗涤至中性,准确量取40 mL 0.1 mol/L HCl加入色谱柱内,收集流出液并用50 mL 1 mol/L NaCl洗涤收集,在流出液中滴加1~2滴酚酞指示剂,用0.1 mol/L NaOH进行滴定,至溶液由无色变为粉色,记录滴定所消耗的NaOH溶液体积,按式(1)计算离子交换容量:


(1) $$IC=\frac{V_{HCl}c_{HCl}-V_{NaoH}c_{NaOH}}{V_{\text{球 }}}$$

式中:*V*_HCl_为HCl溶液体积,mL; *c*_HCl_为HCl溶液浓度,mol/L; *V*_NaoH_为NaOH溶液体积,mL; *c*_NaOH_为NaOH溶液浓度,mol/L; *V*_球_为微球体积,mL。

#### 1.3.2 蛋白静态结合容量(SBC)

将一定量的FastSep-PAA与BSA溶液(该溶液用50 mmol/L pH 8.0的Tris-HCl缓冲液配制)混合,室温吸附6 h,离心后取上清液,用紫外可见分光光度计在280 nm波长下测定其吸收值,通过标准曲线计算剩余蛋白浓度*C*_剩余_,按式(2)计算蛋白静态结合容量:


(2) $$SBC=\frac{\left(C_{\text{初始 }}-C_{\text{粯余 }}\right)V_{BSA}}{V_{\text{球 }}}$$

式中:*C*_初始_为初始BSA溶液的质量浓度,g/L; *C*_剩余_为剩余BSA溶液的质量浓度,g/L; *V*_BSA_为BSA溶液体积,mL。

#### 1.3.3 蛋白动态结合容量(DBC)

将介质装填于聚丙烯色谱柱(*Φ* 7.8 mm×13 mm)中,以50 mmol/L pH 8.0 Tris-HCl缓冲溶液作为平衡缓冲溶液,1 mol/L氯化钠溶液作为洗脱液(该溶液用50 mmol/L pH 8.0 Tris-HCl缓冲溶液配制),先用平衡缓冲液将色谱柱平衡至基线平稳,之后切换进样阀至蛋白溶液(2 g/L BSA溶液)进行液体自动进样,当流穿曲线高度达到最高点后停止进样,先用平衡缓冲溶液冲洗色谱柱至基线重新平稳,然后用洗脱液对蛋白进行洗脱,记录10%流穿体积,按式(3)计算蛋白动态结合容量:


(3) $$DBC=C_{BSA}V_{10\%\text{流穿}}/V_{\text{球}}$$

式中:*C*_BSA_为进样蛋白的质量浓度,g/L; *V*_10%流穿_为10%流穿体积,mL。

### 1.4 介质结构及孔径尺寸测定

介质的表面形貌结构通过SEM进行观察;其比表面积分析采用氮气吸附法测定;采用压汞法对介质孔径尺寸和分布进行表征。

## 2 结果与讨论

### 2.1 反应条件对介质IC及蛋白SBC的影响

#### 2.1.1 PAA浓度

以FastSep-epoxy为基质,通过微球表面的环氧基与PAA分子的伯氨基反应,将PAA分子接枝至大孔微球表面,制备得到FastSep-PAA,其制备过程如图1所示。

**图 1:**
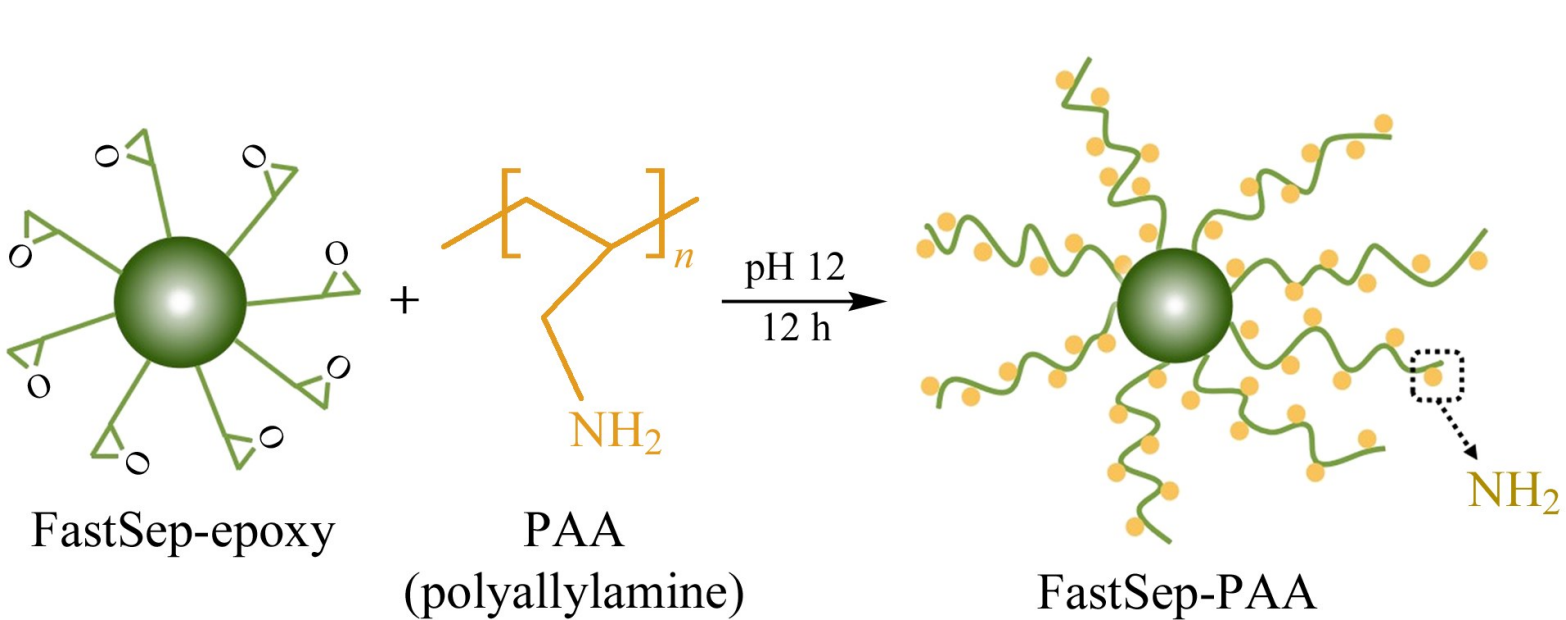
FastSep-PAA的制备过程

介质表面氨基数量决定其表面吸附位点数量,故首先考察了PAA质量浓度(*C*_PAA_)对蛋白静态结合容量的影响,其结果如图2所示。随着*C*_PAA_的增加,IC增加,SBC先增加后不变。在*C*_PAA_小于0.05 g/mL时,IC较低,蛋白吸附位点较少,故SBC较低;增加*C*_PAA_, IC增高,蛋白吸附位点增多,蛋白结合能力增加,*C*_PAA_为0.2 g/mL时SBC达到最高值(101.3 g/L);继续增加*C*_PAA_, IC继续增加,但SBC不再随之增加。

**图 2:**
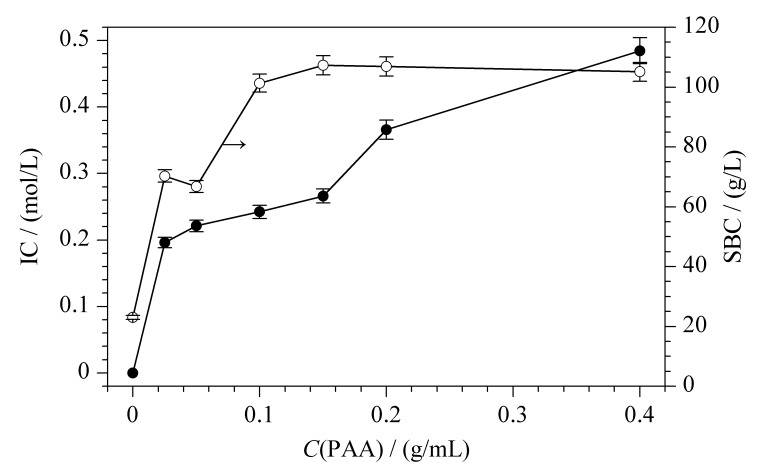
PAA质量浓度对介质IC和蛋白SBC的影响(*n*=3)

类似规律出现在李明等^[19]^的研究中,该研究将17.5 kDa的PAA分别接枝到琼脂糖介质(Sepharose FF)上,制备得到一系列不同IC的阴离子交换色谱介质(Sepharose FF-PAA-S),分别考察了IC对蛋白SBC的影响,发现随IC增加,蛋白吸附位点增加,蛋白SBC增加;此外,链伸展程度增加、链间距减小会使蛋白SBC快速增加,而孔隙体积减小则会使蛋白SBC降低。所以随着*C*_PAA_的增多,微球内部分孔隙体积被占据,空间位阻逐渐增大,蛋白的可及孔隙空间减小^[14,19]^,故随配基密度增加SBC基本保持不变,所以在后续实验中不再继续增加PAA浓度,选择0.2 g/mL为最优单体浓度。

#### 2.1.2 反应时间

反应时间对介质IC及蛋白SBC的影响规律如图3所示,考察范围为0.5~48 h,随着反应时间的增加,IC先增加后逐渐稳定,而SBC则略有下降。根据IC随反应时间的变化趋势,可以将反应时间分为3个阶段(反应初期、反应中期和反应末期)。在反应初期(3 h内),微球表面富含环氧基,PAA的接枝速率快,IC随时间增加而迅速增加(3 h时达到0.31 mol/L);在反应中期(3~12 h),微球表面的环氧基逐渐减少,PAA接枝速率降低,IC随时间增加而缓慢增加(12 h时达到0.37 mol/L);在反应末期(12~48 h),微球表面的环氧基几乎完全被反应掉,PAA不再继续增加,IC随时间增加而基本保持不变(48 h时保持在0.37 mol/L)。随着反应时间的增加,接枝的PAA链不断增多,互相缠绕,造成了微球内部分孔隙体积被占据,孔内空间位阻逐渐增大,蛋白的可及孔隙空间减小^[15]^,故SBC稍有下降(0.5 h时110.5 g/L, 48 h时99.0 g/L)。因此,为了获得最佳的接枝效果,需要选择合适的反应时间,通过比较不同反应时间下的 SBC和IC,找到最佳的反应时间。在接枝PAA单体的过程中当反应时间为12 h时,SBC达到最高,故选择12 h作为最优反应时间。

**图 3:**
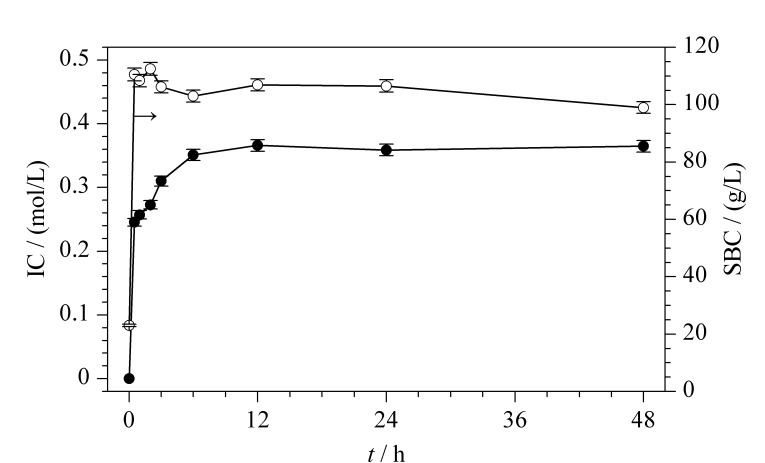
反应时间对介质IC和蛋白SBC的影响(*n*=3)

#### 2.1.3 pH值

反应溶液的pH值影响PAA中氨基的质子化程度,进而影响其反应效率,通过考察pH 7~14范围内IC和SBC的变化(如图4所示),发现二者都随着pH升高先增加后减小,在pH 12时为最大值,IC=0.37 mol/L, SBC=106.86 g/L。pH对环氧基的反应活性和氨基质子化程度有较大影响,其影响规律^[9]^如下:环氧基本身存在较大角张力,性质活泼,易于发生亲核取代反应而开环,在碱性条件下,亲核试剂优先进攻取代较少的碳原子,反应较容易发生;在酸性条件下,亲核试剂优先进攻取代较多的碳原子,反应位阻大从而较难反应。PAA是直链碱性聚电解质,p*K*_a_=9.67^[22]^。当pH<9.67时,PAA链上的氨基质子化,即以-N ${H_{3}}^{+}$ 和-N ${H_{2}}^{+}$ 形式存在,接枝PAA后的微球与游离的PAA之间静电斥力增加,PAA接枝效率低;当pH > 9.67时,PAA链上的氨基脱质子化,即以-NH_2_形式存在,游离的PAA在微球中的扩散不受限制,PAA接枝效率高^[22]^。此外,继续增加pH值,会增加微球上环氧基水解的反应速率^[5]^,导致接枝效率降低,因此存在最优pH值(pH 12)。

**图 4:**
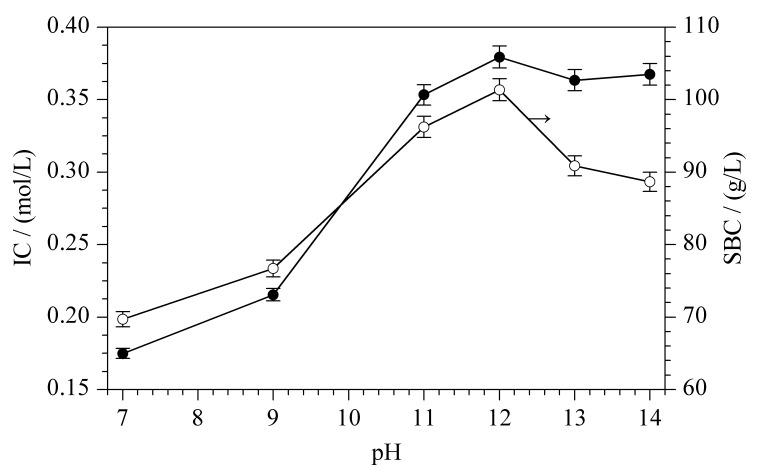
pH对介质IC和蛋白SBC的影响(*n*=3)

### 2.2 色谱介质的表面形貌表征

通过SEM对FastSep-epoxy和FastSep-PAA的形貌进行了表征,如图5所示。从FastSep-epoxy的全貌、表面及内部结构图,可以看出,该微球的表面及内部均存在微米级的大孔,同时孔隙之间有较好的连通性,这些特征均有利于蛋白等生物大分子在介质内部的自由扩散。

**图 5:**
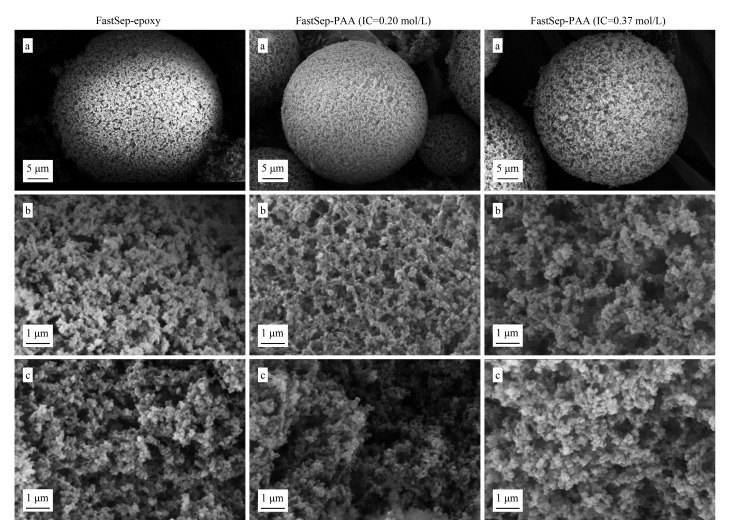
FastSep-epoxy与FastSep-PAA的SEM图

将图5中低IC(0.20 mol/L)与高IC(0.37 mol/L)的FastSep-PAA的全貌、表面及内部结构图与FastSep-epoxy进行对比,发现其接枝前后,微球球形未发生变化,微球表面及内部孔道结构无明显变化,孔道连通性较好,内外部孔径无明显差异,蛋白传质无影响。

### 2.3 色谱介质孔径对蛋白DBC的影响

色谱介质孔径尺寸对于蛋白传质有显著影响^[23,24]^,本工作考察了FastSep-PAA原始孔径尺寸(即FastSep-epoxy的孔径尺寸)对蛋白DBC的影响规律。首先通过压汞法测定FastSep-epoxy的孔径尺寸(厂家标注平均孔径尺寸范围300~1000 nm),其结果如图6所示,测得的平均孔径与微球厂家说明书所给的平均孔径较为相符,因此下文继续按厂家所提供的平均孔径对其进行区分。

**图 6:**
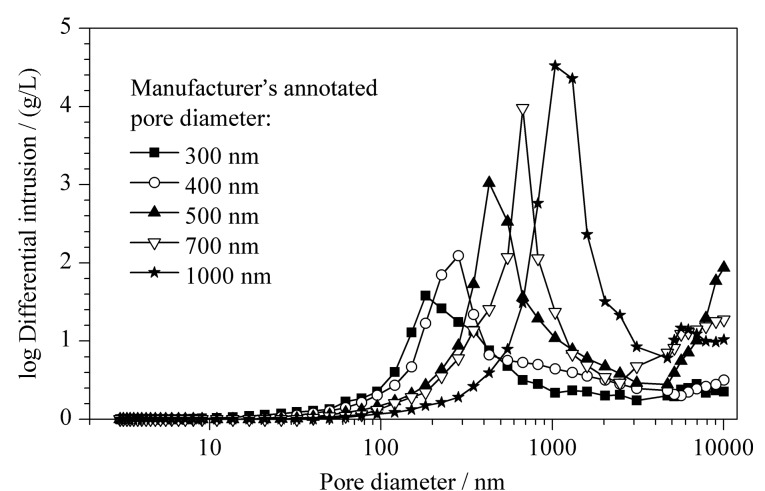
压汞法测定的FastSep-epoxy的孔径分布

对不同孔径的FastSep-PAA色谱介质进行了蛋白DBC测定,其结果如表1所示。在300~1000 nm孔径范围内,其蛋白DBC并未出现随介质孔径尺寸增加而显著下降的现象。其中原始孔径为400 nm的介质蛋白动态结合容量最高(70.3 g/L);原始孔径增大至1000 nm孔径时,其蛋白动态结合容量有所下降,但仍能保持在60.0 g/L水平,这是因为孔径增大有利于提高蛋白在介质内部的传质速度,这抵消了由孔径增大导致比表面积降低带来的部分不利影响;减小原始孔径至300 nm时,FastSep-PAA介质的DBC稍有降低(68.6 g/L),这可能是由于接枝的大分子PAA长链占据了部分孔隙内空间,孔内空间位阻增大,导致蛋白的可及孔隙空间减小,DBC降低^[14,19]^。

**表 1:** 不同原始孔径的FastSep-PAA的DBC

FastSep-epoxy pore size^a^/nm	FastSep-epoxy pore size^b^/nm	DBC/(g/L)
300	324	68.6
400	419	70.3
500	541	61.3
700	717	58.6
1000	1038	60.0

a: FastSep-epoxy average pore size provided by manufacturer’s instructions; b: FastSep-epoxy average pore size by mercury intrusion method.

为更进一步分析接枝PAA对于介质结构的影响,采用氮气吸附法测定了接枝后不同孔径的色谱介质的孔径分布。本工作所用PAA分子的分子质量范围为10~20 kDa,根据文献[25],按式(4)计算PAA的分子半径:


(4) $$r=\frac{k_{B}TM^{0.549}}{4\pi \eta K}$$

式中:*k*_B_是玻尔兹曼常数,1.380649×10^-23^ J/K; *T*是温度,K; *M*是聚合物的相对分子质量;*η*是黏度,Pa·S; *K*=3×10^-8^ m^2^/s,由聚合物-溶剂系统所决定。

将所用的PAA分子的相对分子质量和具体实验环境温度代入公式(4)计算得到的PAA分子尺寸为1.7~2.5 nm,该类分子化学键合至孔道表面后,对介质的介孔(2~50 nm)影响较大,因此,文中采用氮气吸附法主要对介质中小于100 nm的孔径进行分析对比,所测定结果如图7所示。从BET图中可以看出:接枝后的介质FastSep-PAA孔径分布均主要由介孔组成,大孔(>50 nm)含量随孔径增加而增加,而微孔(<2 nm)则几乎没有,这是因为接枝PAA堵塞了微孔^[19,21]^。

**图 7:**
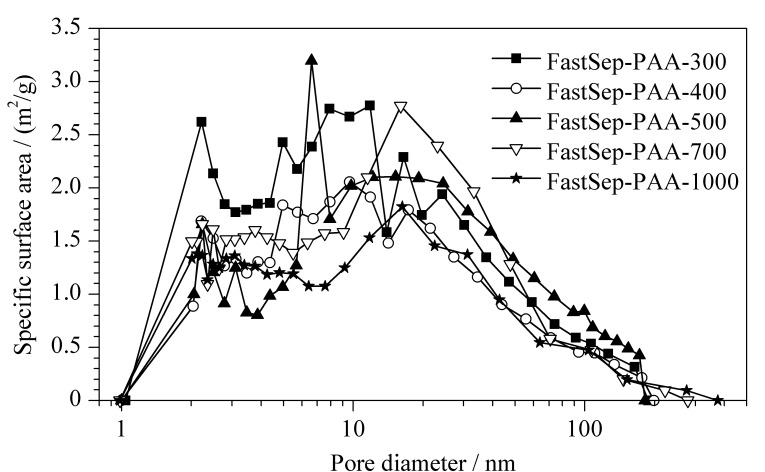
BET测定不同孔径FastSep-PAA的比表面积

图7显示FastSep-PAA-300、400、500、700和1000孔径分布主要集中于2~10、5~20、10~40、10~50和10~60 nm。对比PAA分子尺寸发现,FastSep-PAA-300结构受PAA接枝影响最大,PAA的接枝会导致介质孔隙内空间减小,孔内空间位阻增加,导致蛋白的可及孔隙空间减小,从而造成了蛋白结合容量的降低^[19,21]^;而其他孔径介质结构虽然也受PAA接枝影响,但孔径增加有利于提高蛋白分子在介质内部的传质速率,这一效应有利于提高蛋白结合容量,这抵消了接枝PAA带来的不利影响,因此出现了DBC随孔径增加而不变的情况。

### 2.4 流速对色谱介质的蛋白DBC的影响

介质孔径增加通常有利于蛋白分子在其内部传质^[2,7,26]^。为了进一步验证2.3节中的孔径尺寸影响蛋白传质的结果,分别测定不同孔径的介质在63、126、251、377、502、628 cm/h流速下的蛋白DBC,不同孔径的色谱介质的蛋白DBC随流速变化如表2所示。在相同流速下,比较不同孔径介质的蛋白DBC, FastSep-PAA-400均表现出较高的蛋白DBC,这是因为相比较其他较大孔径的介质,该孔径比表面积较大,蛋白可吸附位点较多;而FastSep-PAA-300接枝PAA后孔隙内空间减小,孔内空间位阻增加,蛋白可及孔隙空间减小,DBC下降,这抵消了比表面积增大带来的部分优势^[19,20]^。

**表 2:** 不同原始孔径的FastSep-PAA在不同流速下的DBC

Original pore size^a^/nm	DBC under different flow rates/(g/L)						Decrease in DBC^b^/%
	63 cm/h	126 cm/h	251 cm/h	377 cm/h	502 cm/h	628 cm/h	
300	68.7	68.6	60.1	59.4	65.5	58.7	14.4
400	76.4	70.3	62.9	63.3	59.7	56.3	19.9
500	68.0	61.3	62.1	58.1	56.3	52.9	13.7
700	68.2	58.6	60.3	55.9	51.0	46.5	20.6
1000	56.9	60.0	62.9	62.3	59.7	57.7	3.5

a: FastSep-epoxy average pore size was provided by manufacturer’s instructions. b: The difference in protein DBC on chromatographic media with different pore sizes were calculated at flow rates of 126 cm/h and 628 cm/h, respectively.

在较小原始孔径(300、400、500、700 nm)的FastSep-PAA介质的蛋白DBC测试中,随着流速的增加,蛋白的穿透体积减小,蛋白DBC下降,比较126 cm/h和628 cm/h流速下的蛋白DBC,流速增加5倍,蛋白DBC下降幅度均在13%~21%,这是因为介质本身孔径就相对较小,而且吸附的蛋白也会占据部分孔隙内空间,造成孔内空间位阻增加,甚至堵塞孔道,因此随着流速的增加,越来越多的蛋白分子来不及扩散进入介质内部,造成了DBC的下降^[2,9]^;而在较大原始孔径(1000 nm)的FastSep-PAA介质测试中,随着流速的增加,蛋白DBC降低幅度较小(57~63 g/L),流速增加5倍,蛋白DBC仅下降3.5%,这说明蛋白分子在该孔径下的扩散速率受限较小^[2,9]^,该孔径介质的传质性能优异,适合于高通量分离纯化,而且在628 cm/h的流速下蛋白DBC能够保持在57.7 g/L。

## 3 结论

本工作以大孔聚丙烯酸酯微球为基质,通过接枝聚(烯丙基胺)制备了大孔阴离子交换层析介质。通过精确控制接枝过程中的反应条件,进一步优化接枝效率,获得了具有高性能的介质;进一步探究了孔径、流速对蛋白动态结合容量的影响,结果表明FastSep-PAA-1000介质的蛋白传质性能优异,在高通量分离纯化中具有较好的应用前景。
